# PCR-Based Equine Gene Doping Test for the Australian Horseracing Industry

**DOI:** 10.3390/ijms25052570

**Published:** 2024-02-22

**Authors:** Tessa Wilkin, Natasha A. Hamilton, Adam T. Cawley, Somanath Bhat, Anna Baoutina

**Affiliations:** 1 National Measurement Institute, Lindfield, NSW 2070, Australia; 2 Faculty of Veterinary Science, University of Sydney, Camperdown, NSW 2006, Australia; 3 Equine Genetics Research Centre, Racing Australia, Sydney, NSW 2000, Australia; 4 Australian Racing Forensic Laboratory, Racing NSW, Sydney, NSW 2000, Australia; 5 Racing Analytical Services Limited, Flemington, VIC 3031, Australia

**Keywords:** gene doping, real-time PCR, horseracing

## Abstract

The term ‘gene doping’ is used to describe the use of any unauthorized gene therapy techniques. We developed a test for five likely candidate genes for equine gene doping: *EPO*, *FST*, *GH1*, *IGF1,* and *ILRN1*. The test is based on real-time polymerase chain reaction (PCR) and includes separate screening and confirmation assays that detect different unique targets in each transgene. For doping material, we used nonviral (plasmid) and viral (recombinant adeno-associated virus) vectors carrying complementary DNA for the targeted genes; the vectors were accurately quantified by digital PCR. To reduce non-specific amplification from genomic DNA observed in some assays, a restriction digest step was introduced in the PCR protocol prior to cycling to cut the amplifiable targets within the endogenous genes. We made the screening stage of the test simpler and faster by multiplexing PCR assays for four transgenes (EPO, FST, IGF1, and ILRN1), while the GH1 assay is performed in simplex. Both stages of the test reliably detect at least 20 copies of each transgene in a background of genomic DNA equivalent to what is extracted from two milliliters of equine blood. The test protocol was documented and tested with equine blood samples provided by an official doping control authority. The developed tests will form the basis for screening official horseracing samples in Australia.

## 1. Introduction

The Australian horseracing industry is the second largest Thoroughbred racing and breeding industry internationally, contributing over $9.1 billion Australian dollars (AUD) value to the national economy [1]. At least one third of the contribution to this figure comes from wagering. For consumers to invest in wagering with confidence, it is essential to maintain a fair gambling environment. Any factor that can potentially impact the integrity of racing could be expected to impact wagering, and, thus, the funding of both the industry and the Australian economy.

Doping control is central to maintaining integrity of horseracing. A major challenge for anti-doping authorities and scientists is the anticipation of future doping trends, with the pharmaceutical industry inadvertently providing an endless source of novel potential doping substances. Developments in the field of genetic therapies have opened new opportunities not only for people suffering from serious disease, but also to those looking for new, undetectable ways to improve physical performance. The use of genetic manipulation to enhance performance has been termed ‘gene doping’ and is prohibited by the World Anti-Doping Agency (WADA) [2], the International Federation of Horseracing Authorities [3], and the Fédération Equestre Internationale [4], the internationally recognized industry bodies that oversee the governance of human sports, horseracing, and equestrian events.

Apart from threatening wagering integrity, violating the spirit of sport, and challenging the principles of fair play, gene doping also presents serious health and welfare risks to equine athletes (reviewed in [5,6]). Human gene therapy trials and pre-clinical studies in animals revealed that adverse effects, including the development of cancer and very rare fatal outcomes, can be associated with expression of specific genes, induction of immune responses to gene delivery or expression, transgene integration into the host genome with oncogenic effects, generation of replication competent virus, or side effects associated with the quality of the vector preparation or the procedure of vector administration. Of particular relevance to equine athletes is the recent study in a single Thoroughbred horse that, following administration of a human EPO transgene, developed anemia [7].

For more than fifteen years, detection strategies to combat gene doping in human athletes have been the subject of significant research (reviewed in [8]). Several groups have focused on developing direct detection approaches to target the exon–exon junctions that are a unique feature of transgenes based on coding DNA (cDNA) for the doping gene. These approaches include different types of polymerase chain reaction (PCR) (end-point, nested, real-time, and digital) and multiple parallel sequencing (MPS) [9,10,11,12,13,14,15,16,17]. Real-time PCR is an ideal method for a commercial gene doping detection test because of its high sensitivity and specificity, speed, simplicity, and accessibility.

Subsequently, real-time and digital PCR have formed the basis of the first generation of equine gene doping detection methods that target a single or multiple transgenes in one reaction [7,18,19]. The utility of real-time PCR has also been demonstrated by the development of assays that target vector sequences rather than inserted transgenes [20]. More recently, MPS has also been evaluated as a gene doping detection approach in racing horses [21,22]. Despite many advantages, this method, as well as those that are based on digital PCR (dPCR) and microfluidic PCR, uses instrumentation that is not available in a conventional equine doping testing laboratory.

To fulfil a requirement to develop an equine gene doping detection program to focus on the large Thoroughbred horseracing population in Australia, we aimed to develop a reliable, easy to use, and economical real-time PCR-based test for gene doping in Thoroughbreds, targeting five genes that we considered highly probable candidates for this new form of doping.

This paper describes the development of PCR assays to detect the cDNA-based transgenes for the erythropoietin (*EPO*), follistatin (*FST*), growth hormone 1 (*GH1*), insulin-like growth factor-1 (*IGF1*), and interleukin-1 receptor antagonist (*IL1RN*) genes in horses. It also describes the evaluation of their fitness for purpose in a blind testing of mock positive and negative equine blood samples. We also present how we applied the test to blood samples collected from racehorses out of competition as part of the normal surveillance testing program carried out over a racing carnival.

## 2. Results

### 2.1. PCR Assay Design

A typical doping detection test consists of two stages, namely screening and confirmation, which rely on complementary methods to detect the doping agent to make the test legally defensible. To meet this requirement, for each doping gene (further referred to as a transgene), we designed two PCR assays that detect different unique exon–exon junctions within the cDNA of the gene.

The assay design was performed using the Beacon Designer software. We aimed to target the primers to adjacent exons within cDNA, while the probe was targeted to the exon–exon junction. In one assay, a suitable probe could not be designed spanning an exon–exon junction, so the junction was targeted by a primer. All primers and probes were designed to avoid targeting areas of known sequence variation.

Multiple assays were designed for the five transgenes and evaluated using various bioinformatics software, as listed in Baoutina, 2016 [23]. Ten assays with the best in silico characteristics were selected (Table 1 and Table 2). The sequences of all but two assays’ oligonucleotides are not presented here to preserve the integrity of the gene doping test but can be made available on request to the corresponding author after signing a non-disclosure agreement.

### 2.2. PCR Assays Evaluation and Optimization in Simplex

To develop and evaluate a test for the five candidate doping genes, we used an in vitro system that imitates a DNA extract from an equine plasma sample, where circulating genomic DNA and transgenes are co-extracted. As the doping material, we used nonviral (plasmid, pDNA) and viral (recombinant adeno-associated virus, rAAV) vectors that incorporate doping genes (refer to Section 4). Equine genomic DNA (egDNA) was sourced commercially. Both vectors and egDNA were accurately quantified by dPCR.

Each assay was optimized in simplex to detect their corresponding transgene with high specificity and sensitivity. The optimized primers concentration in all assays was 600 nM. The optimal probe concentration was 200 nM, except for the screening *FST* assay and both *IGF1* assays, where it was 150 nM. All screening assays performed best at the annealing/extension temperature of 60 °C. For confirmation assays, the optimal annealing/extension temperature was 56 °C (FST, GH1, and IGF1 assays) or 58 °C (EPO and IL1RN assays). The duration of the annealing/extension step for all assays was 30 s. For each assay, the fluorescence threshold (dR, baseline corrected raw fluorescence) was manually determined, validated, and fixed for future analyses (refer to Section 4).

**Figure 1:**
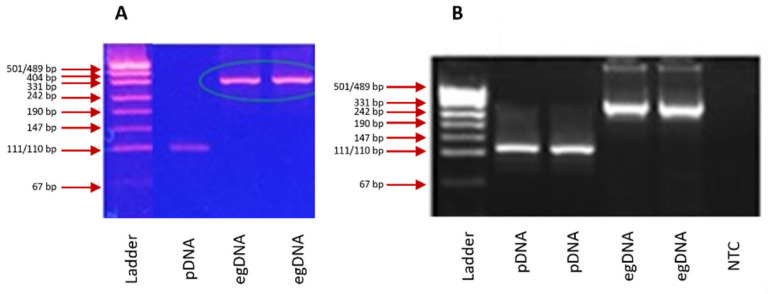
Fragment size analysis (FSA) of products amplified from the GH1 transgene (marked pDNA) and genomic DNA (marked egDNA) in GH1-specific screening (**A**) and confirmation (**B**) assays. In both assays, the amplicons generated from the transgene are of the expected sizes (109 bp (**A**) and 120 bp (**B**), Table 2). The sizes of amplicons generated from egDNA correspond to amplicons from the endogenous *GH1* gene (308 bp (**A**) and 333 bp (**B**)).

The assays’ specificity was confirmed by analysis of the sequence, size, and melting temperature of the generated amplicons, and by testing negative controls, which included negative template control (NegTC) and no template control (NTC). The NegTC (egDNA at 100 ng/well; refer to Section 4.4) returned negative PCR results in all assays and in all analyses performed. Post-PCR gel electrophoresis (further referred to as fragment size analysis, FSA) revealed that the two GH1 assays (Figure 1) and the confirmation assays for the EPO and FST transgenes generated non-specific products. The sizes of these products indicated that amplification occurred from the corresponding endogenous genes (Table 2) because of a relatively short intron flanked by the exons targeted by the primers. Importantly, since the endogenous genes do not contain exon–exon junctions that are targeted by the assays’ probes, these non-specific products were not detected in real-time PCR, and no false-positive results were recorded. FSA of NegTC in the EPO screening assay showed a faint band of a smaller size (approximately 67 bp) than the size of the transgene-specific amplicon (100 bp). This band could not be eliminated despite extensive assay optimization and appeared to be too large to be caused by a primer dimer. However, since no false-positive PCR result was detected in any replicates of NegTC in the EPO screening assay, further investigation of the identity of this product was not performed.

**Figure 2:**
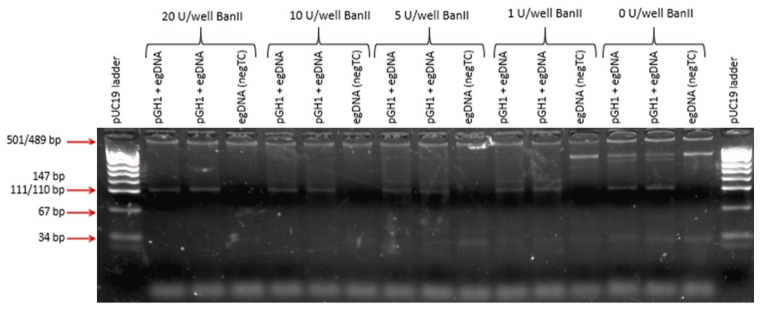
Fragment size analysis (FSA) of products generated in the GH1 screening PCR assay preceded by restriction digest with BanII at different amounts (20, 10, 5, and 1 U/well). Each reaction (PCR well) contained 100 ng egDNA with or without 10 copies of plasmid carrying the GH1 transgene (pGH1). The bands for the amplicon from the transgene and the endogenous *GH1* gene correspond to 109 bp and 308 bp, respectively, as expected (Table 2).

Generation of non-specific amplicons from genomic DNA, even if not detected in real-time PCR, may affect amplification efficiency for the transgene by consuming PCR reagents, especially when the transgene is present in the sample at a very low concentration. To minimize amplification of egDNA, we looked for a restriction enzyme (RE) that would cut the endogenous gene within the amplifiable intron and be effective in the PCR master mix. For restriction digestion with the GH1 screening assay, BanII enzyme best met our requirements. When the enzyme was added to PCR mix at the optimum concentration of 5 U/well and a pre-cycling digestion step (37 °C for 60 min) was added in the thermal profile, amplification of the endogenous *GH1* gene was prevented (Figure 2). Since we planned to multiplex the screening assays, we tested and confirmed that the presence of BanII at 5 U/well did not affect the screening assays for other transgenes. Subsequently, all screening assays were performed in the presence of BanII with the thermal profile that included the pre-cycling digestion step.

For the EPO, FST, and GH1 confirmation assays, MspI, BanII and EcoRI, respectively, were shown to be the most suitable restriction enzymes. Their optimal concentration was found to be 10 U/well. The use of EcoR1 in the GH1 assay eliminated amplification from the endogenous gene, while the use of a RE in the EPO and FST assays significantly reduced, but did not eliminate, generation of a non-specific product from the endogenous gene. Since the IGF1 and IL1RN confirmation assays did not generate non-specific products from the endogenous genes, the reaction mixes and thermal profiles for these assays remained unchanged.

### 2.3. PCR Assay Evaluation in Simplex

The assays were evaluated and validated using key PCR performance parameters including amplification efficiency and linearity (the squared correlation coefficient *R*^2^), specificity, sensitivity, and dynamic range, following the recommendations of ISO 17025 [24] and similarly to validation of PCR methods performed by us and others [7,19,25,26].

Standard curve analysis for each transgene in pDNA was initially performed in Agilent Technologies Brilliant II qPCR master mix (Supplementary Table S1). Since we planned to perform the screening stage of the test in multiplex, the same conditions were subsequently tested in Qiagen qPCR Multiplex master mix (further referred to as Qiagen Multiplex MM). Qiagen Multiplex MM showed improved sensitivity of the assays in all conditions (Supplementary Table S1). Based on these results and having confirmed that all three restriction enzymes are active in this master mix, Qiagen Multiplex MM was chosen for both stages of the test and was used from then on.

For all ten assays analyzed with pDNA in a TE_0.1_ buffer, amplification efficiency was close to 100% (between 96 and 105%) and linearity was close to 1 (between 0.96 and 1) over the dynamic range of five orders of magnitude (Supplementary Table S1). In the presence of egDNA at 100 ng/well, amplification efficiency and linearity in some assays deviated from the ‘theoretical ideal’ values of 100% and 1, respectively. The lowest efficiency was 69% for the GH1 confirmation assay, which improved to 92% with the addition of the pre-PCR restriction digest step. The lowest linearity was 0.91 for the EPO confirmation assay.

Next, we compared transgene cycle threshold (Ct) values in different conditions. In real-time PCR, the cycle threshold is defined as the cycle at which the fluorescence from the reaction crosses a specified threshold level at which the signal can be distinguished from background levels [27]. The average Ct value for the lowest dilution analyzed (10 copies (cp)/well) (*n* = 2) was slightly higher (up to 1.6 Ct values) in the presence of egDNA than in its absence in all screening assays and in confirmation assays for the FST, IGF1, and IL1RN transgenes (eight assays in total). In the EPO and GH1 confirmation assays, their target transgene at 10 cp/well was undetectable in all (EPO assay) or most (GH1 assay) replicates tested. When the pre-PCR restriction digest step was included to reduce non-specific amplification, the assays’ performance improved. In the eight assays mentioned above, the average Ct value (*n* = 2) for the lowest amount of pDNA tested in the presence of the appropriate RE decreased by between 0.4 and 4.9 cycles, while in the EPO and GH1 confirmation assays, all replicates were detected with an average Ct of 34.6 ± 0.1. Since these conditions (Qiagen Multiplex MM and RE digest of pDNA prior to PCR) produced the most sensitive detection of the target transgenes in pDNA, they were used in all subsequent experiments.

Next, the standard curve analyses were performed with the doping genes in a viral vector (rAAV) in the presence of egDNA (100 ng/well). Amplification efficiency and linearity of all assays remained close to, respectively, 100% and 1. Both duplicates of the lowest amount of rAAV tested (20 viral genomes per well) were detected.

The minimum amounts of doping genes that could be reliably (in ≥95% replicates) detected by the assays are shown in Table 3. For the FST, GH1, IGF1, and IL1RN screening assays and all confirmation assays, the lowest concentrations of transgenes reliably detected was 10 cp pDNA and 10 viral genomes (vg) rAAV, except for the GH1 screening and IL1RN confirmation assay, where 20 cp pDNA and 15 vg rAAV were detected in more than 95% replicates. The EPO screening and confirmation assays reliably detected 20 cp pDNA and 20 vg rAAV.

The assays were also tested for the detection of a fixed amount of the transgene in pDNA (160 cp/well) in the presence of increasing amounts of genomic DNA (Supplementary Figure S1). For most assays, Ct values were generally stable in the presence of up to 200 ng of egDNA. In the presence of 500 ng egDNA, the Ct values in all assays increased, although in the confirmation assays the increase was lower than in the screening assays. This amount of egDNA in PCR in a real gene doping testing scenario using blood plasma as DNA extraction matrix is, however, highly unlikely, since the average DNA concentration in DNA extracts from equine plasma that we measured was 25 ng/µL, resulting in 100 ng egDNA per PCR well.

### 2.4. PCR Assay Evaluation in Multiplex

The screening assays optimized in simplex were combined in various multiplex combinations and their performance was tested as described in Section 4. The results showed that the EPO, FST, IGF1, and IL1RN assays performed similarly to when they were used in simplex, while the screening GH1 assay was not as efficient in any multiplex format as it was in simplex.

In five-plex format, the EPO, IGF1, and IL1RN transgenes were detected only in their corresponding detection channels. When the GH1 transgene was analyzed, in addition to a positive PCR result in the Cy3 channel, amplification was detected in the JOE channel in one of two replicates. Similarly, when the FST transgene was analyzed, in addition to a positive PCR result in the JOE channel, amplification was detected in the Cy3 channel in both replicates. To investigate the origin of amplification, the PCR products from the false-positive reactions were analyzed by gel electrophoresis. Since no evidence of cross-contamination with another target was obtained, we concluded that crosstalk between these two channels (Supplementary Figure S2) was the likely cause of the observed false-positive PCR signals.

Based on the suboptimal performance of the screening GH1 assay in any assay combination and the observed crosstalk between the channels that detected the GH1-specific probe (Cy3) and FST-specific probe (JOE), we chose to use a four-plex combination of the EPO, FST, IGF1, and IL1RN assays and a separate simplex GH1 assay in the screening stage of the test.

### 2.5. Validation of the Screening Multiplexed Panel for Detecting the EPO, FST, IGF1, and IL1RN Transgenes

Validation of the four-plex panel was performed similarly to validation of each assay in simplex. Transgenes were amplified in the range of 10 to 10^5^ cp/well for pDNA or 20 to 2 × 10^5^ vg/well for rAAV in the presence of background egDNA at 100 ng/well and including pre-PCR restriction digest with BanII, as described earlier. Amplification efficiency for each transgene was close to 100%, with the lowest and the highest efficiency being, respectively, 92% for rAAV-IL1RN and 107% for rAAV-FST. Linearity for detecting each target by the four-plex assay panel was close to 1, ranging between 0.96 and 1. These results were comparable to those obtained when each transgene was analyzed by its specific assay in simplex (Supplementary Table S1). For the FST, IGF1, and IL1RN transgenes, the lowest concentrations that were detected in the four-plex panel with 95% confidence were 10 cp/well for pDNA and 20 vg/well for rAAV (10 vg rAAV was not tested in the multiplex format) (Table 4). The sensitivity of detecting the *EPO* transgene in the four-plex format with 95% confidence was the same as in simplex (20 cp/well) for pDNA and slightly lower for rAAV (40 vg/well).

All NegTC and NTC (*n* = 10 for both) analyzed in the four-plex screening panel were PCR negative in all four channels, confirming the high specificity of the test.

We also assessed the detection capability of the four-plex assay panel with multiple targets present in the sample. For that, up to four targets in pDNA were added in one PCR, each at 160 cp/well in the presence of egDNA at 100 ng/well. Each transgene was detected in its corresponding channel with a Ct value that was similar to when this transgene alone was present in the reaction mix, with the exception of the EPO transgene. When EPO was mixed with either the FST or IL1RN transgenes, its Ct increased by 3.0 and 1.8 cycles, respectively. These results indicate that if more than one transgene from the group of four (EPO, FST, IGF1, and IL1RN) is used for doping, the screening four-plex panel should detect them with similar reliability as when a single transgene is used for doping.

### 2.6. Controls

The quality controls include positive and negative extraction controls (PEC and NEC, refer to Section 4.7), a positive template control (PTC, 160 cp of the corresponding pDNA/well), and a negative template control (NTC) for each assay.

To test for possible PCR inhibition by impurities in the DNA extract (‘matrix effect’) and to alert researchers to possible false-negative results, the inhibition control (IC) for each sample is also analyzed. The IC wells contain half the volume of the sample DNA extract that is used in PCR analysis of the sample, and half the amount of pDNA used in PTC (80 copies). PCR inhibition is assessed based on two parameters: the IC’s Ct value (the average of PCR duplicates) and the difference between this value and the Ct value for PTC (also the average of PCR duplicates) in the assay. We use the term ‘delta Ct’ for this difference. Since the IC contains half the amount of DNA template compared to the PTC, if PCR amplification is not inhibited, the IC’s Ct value should be around one cycle lower than that of the PTC. The higher the IC Ct value and the delta Ct value, the stronger the inhibition. We classify the degree of inhibition as ‘strong’ if the Ct value for the IC is over 40 and the delta Ct value is greater than 4.

### 2.7. Generating Quality Control (QC) Charts for Positive Controls

To monitor the performance of the screening and confirmation PCR assays, QC charts were generated for PTCs for each assay and each transgene. A typical example of the QC chart for the EPO screening assay is shown in Supplementary Figure S3. The chart plots the Ct value for PTC (the average and variance of PCR duplicates) obtained every time it is analyzed in the transgene-specific assay, together with the calculated historical average Ct value and upper and lower control limits. The latter are calculated as the average historical value plus or minus three standard deviations of all collected Ct values. The PTC QC charts for each transgene generated during this study confirmed that our measurement system was stable over time.

To monitor extraction efficiency and possible errors in the extraction process, the QC chart was also generated for PEC. The average PEC Ct value and control range for PEC analyzed on 11 occasions was 33.1 ± 1.8, confirming good reproducibility of the DNA extraction procedure.

### 2.8. Testing of the Developed Gene Doping Detection Method on Equine Blood Samples

Next, the developed gene doping method and the test protocol were evaluated for their fitness for purpose in two separate studies with equine blood samples. In the first study, blind testing of mock positive and negative samples was performed. The experience gained from this study was used to make improvements to the workflow and the protocol, which were adopted in the second study, that further explored the practicability of the test. This second (‘surveillance’) study was performed on out of competition blood samples collected from racehorses as part of the normal surveillance testing program carried out by Racing NSW.

#### 2.8.1. Blind Testing of Spiked Equine Blood Samples

The preparation, processing, and testing of mock positive and negative for gene doping samples is described in Section 4. PCR analysis followed the test protocol described. For each transgene, two positive blood samples were prepared, one spiked with transgene-specific pDNA (1500 cp/mL) and the other with transgene-specific rAAV (1500 vg/mL).

The FST, GH1, IGF1, and IL1RN screening assays (GH1 in simplex, and FST, IGF1, and IL1RN as part of the four-plex panel with the EPO assay) correctly detected their relevant doping genes in each sample (Table 5). These results were verified by the corresponding confirmation assay. The EPO screening assay correctly identified one sample spiked with the *EPO* transgene, while the second spiked sample was PCR-negative, but was noted to have significant PCR inhibition in the EPO screening assay. This demonstrates the value of the PCR inhibition control in alerting the analyst to the possibility of a false negative. In total, ten of the fourteen samples analyzed in the EPO screening assay showed significant inhibition that indicated the possibility of false-negative findings. No significant matrix effect was detected in other assays. In the case of a real gene doping testing, significant PCR inhibition will require repeat PCR, repeat DNA extraction, and/or further method optimization and validation, for example, the development of new interpretation and acceptance criteria for the IC. Modifications to the test protocol to reduce the ‘matrix effect’ on PCR could also be considered. This could involve addition of DNA cleanup or using a different DNA extraction method that results in higher purity DNA extracts. Any modifications to the protocol will require additional validation. We consider this work for future research.

#### 2.8.2. Surveillance Testing of Samples Collected for Official Doping Testing

Eighteen samples were analyzed for surveillance testing in three batches. Positive and negative controls passed their acceptance criteria. All samples were negative for all five doping genes in the screening stage of the test (Supplementary Table S2 for Batches 1 and 2, and Table 6 for Batch 3). Two samples in Batch 3 showed a significant matrix effect in the GH1 screening assay, invalidating the analysis, and deeming test results for GH1 inconclusive (Table 6). The significant inhibition effect could have been caused by increased hemolysis during sample collection and transportation, or by unknown characteristics of the animals they were taken from. Nonetheless, since all samples returned negative findings or significant inhibition in the screening assays, confirmation testing was not performed.

## 3. Discussion

In this study, we describe the development of a two-stage test protocol for direct PCR-based detection of five equine doping genes, namely *EPO*, *FST*, *GH1*, *IGF1*, and *ILRN1*, which are considered among the most likely candidates for gene doping in racing horses. To our knowledge, the described test is the first published test to target *ILRN1.* The proteins produced from the EPO, FST, GH1, and IGF1 transgenes have the potential to directly enhance performance in racing horses (reviewed in [6]). EPO can improve endurance through increased production of red blood cells and oxygen delivery to tissues, while GH1, IGF1, and FST could have anabolic effects; in the case of FST, this would occur through inhibiting myostatin, a negative regulator of muscle growth. The protein produced from the *IL1RN* gene, interleukin-1 receptor antagonist protein (IRAP), inhibits the inflammation triggered by interleukin-1, thus modulating the inflammatory response [28]. IRAP is a commonly used therapy in horses, primarily as an anti-inflammatory treatment for joint disease. Further, *IL1RN* was one of the first genes tested in pre-clinical trials of gene therapy in horses and is still popular 20 years later (reviewed in [29]). A therapy that could deliver ongoing anti-inflammatory effects that help a horse recover from training and racing more quickly would be very attractive to most trainers. However, if the horse does not need this therapy or its use is not documented as per the requirements of the horseracing authorities, the use of the *IL1RN* would be considered cheating. Thus, we placed high importance on being able to detect doping with this gene. The test developed in this study is the first to target the *IL1RN* gene.

To detect trace amounts of a cDNA-based transgene in a background of its endogenous counterpart in genomic DNA, assays were designed to detect sequences within the cDNA that correspond to exon–exon junctions that are unique to transgenes.

Each assay was optimized and validated using an in vitro system that simulates DNA plasma extracts prepared from equine blood samples. To make screening for gene doping faster and easier, we developed a multiplex format of screening PCR assays for four transgenes (EPO, FST, IGF1, and IL1RN). Screening for the GH1 transgene is performed on its own since its assay showed suboptimal performance when combined with assays for other transgenes. When a sample shows a positive PCR result for a particular gene from screening, the confirmation assay for the gene is performed in simplex. This two-step test protocol that detects different targets within the doping material makes the gene doping test more legally defensible and has already been approved for gene doping detection in human athletes [30]. The use of two assays targeting different exon–exon junction within the transgene has also been shown to be beneficial in equine gene doping testing to avoid potential false-positive results with a single assay [18,19].

During assay validation, we observed that, in the presence of background equine genomic DNA, amplification efficiency, specificity, and sensitivity for some assays decreased. Restriction digestion was tested with the aim to improve assays’ performance as previously demonstrated [12,25]. To reduce non-specific amplification in the four assays that occurred across a short intron framed by targeted exons and only detectable by post-PCR FSA, the pre-PCR digestion of the endogenous gene within the intron was introduced. Importantly, we showed the digestion to be effective in the PCR buffer, allowing this step to be performed in the assembled PCR reaction mix immediately prior to cycling. Subsequently, the PCR protocol for all screening assays and for the EPO, FST, and GH1 confirmation assays was modified to add a suitable RE to the reaction mix and to include a pre-cycling digestion step (37 °C for 60 min) in the thermal profile. This modified one-step protocol simplifies testing and reduces the potential for contamination when digestion and PCR are performed in two separate steps. It also prevents a likely decrease in the sensitivity of transgene detection due to dilution of sample DNA if the digestion step is performed separately.

It should be noted that the non-specific amplification of endogenous genes resulting in the generation of longer amplicons containing an intron that we observed in some assays will be unlikely to have a significant detrimental effect on test performance when blood plasma is used as the DNA extraction matrix. This is because our experimental system consisted of an aqueous solution of a transgene-carrying vector and commercial mostly high molecular weight egDNA as a source of endogenous genes. Plasma is likely to contain fragmented cell-free DNA (from cells undergoing apoptosis and necrosis) and, possibly, low levels of high molecular weight genomic DNA from nucleated blood cells released during blood collection, storage, and processing. The benefit of the restriction digest will likely be more significant when the blood used in the test has undergone prolonged storage or harsh processing, or when whole blood is used as a DNA extraction matrix, as we intend to test in our future work.

An added advantage of a pre-PCR restriction digest is likely to be linearization of a transgene-carrying plasmid, since plasmid linearization is known to improve PCR performance [31,32,33]. The site for BanII in all screening and the FST confirmation assays is located within the EPO, FST, and GH1 cDNA, but outside of their assays’ targets. Therefore, for these transgenes, BanII will linearize any plasmid and remove potential conformational constraints in viral constructs, regardless of the vector sequence. The IGF1 and IL1RN cDNA do not feature a BanII site; thus, plasmid linearization in their screening assays would be achieved only if the plasmid backbone contains a restriction site for this enzyme, as was the case in this study. Incidentally, these two screening assays were least affected by the presence of genomic DNA. Thus, it would be expected that detection of the IGF1 or IL1RN transgenes using the screening assays will be reliable regardless of whether the plasmid is linearized or not. Similarly, the confirmation assays for these transgenes were least impacted by genomic DNA, so their protocols did not include a restriction digest. The observed improved performance of the EPO confirmation assay in a background of genomic DNA by pre-PCR restriction digest with MspI could be the result of a combination of decreased non-specific amplification from the endogenous gene and plasmid linearization, since the EPO-plasmid used in this study features multiple MspI restriction sites. In a real scenario of gene doping, the performance of this assay may change if the plasmid used lacks a recognition site for MspI. On the other hand, the observed benefit of pre-treatment with EcoRI for the GH1 confirmation assay in the presence of genomic DNA was likely attributed to preventing non-specific amplification, since the GH1-plasmid used lacks a multiple cloning site and does not feature the EcoRI site. However, if a plasmid used in a gene doping attempt contains the EcoR restriction site, the performance of this assay is likely to improve further.

We also demonstrated that the PCR master mix has an impact on assay performance. This observation was not surprising, since we (unpublished) and others (for example, [34,35]) have observed this phenomenon, likely attributed to the different polymerases and other components in each master mix.

The PCR-based gene doping detection method developed in this study was shown to have high specificity, as was confirmed by the absence of false-positive results for any of the assays across the validation experiments. The lack of false positives also reflects the merit of stringent laboratory procedures that prevented carryover contamination.

The developed test is highly sensitive, as it is able to detect as few as 10 copies of most transgenes per reaction for the in vitro system that simulates DNA extracts from 1 mL of equine blood plasma. Importantly, the accuracy of the sensitivity values determined is reliant on accurate dPCR-based quantification of each vector used. The FST, GH1, IGF1, and IL1RN transgenes were reliably detected in both stages of the test at 10 copies in the nonviral vector and between 10 and 20 vg in rAAV. Detection of the EPO transgene in the multiplexed screening assays had slightly lower sensitivity, although it was reliably detected at 10 cp in pDNA and 10 vg rAAV in the confirmation assay. Amplification of a short non-specific product from egDNA in the EPO screening assay may contribute to its slightly lower sensitivity, and we will consider focusing future efforts on identifying this product to prevent its amplification. Future test validation with more replicate measurements will identify the absolute limit of detection for each transgene either in a sample alone or in a mixture with other transgenes from the targeted panel. The demonstrated ability of the developed four-plex screening PCR to detect several doping genes in the sample presents an added advantage of the developed test.

The sensitivity of the assays developed in this study is comparable to that of recently published methods for equine gene doping detection. In one study, the limit of detection was estimated to be 6.25 transgene copies per reaction [19]. This slightly lower detectable number of transgenes is likely due to the inclusion of the pre-amplification step in the PCR protocol and the lower amount of genomic DNA (12.5 ng) per reaction used. The sensitivity of our method was similar to that obtained in another study [18], where seven copies of the EPO doping gene per 20 µL reaction were detected by a dPCR method in the presence of 10 ng/µL egDNA. The limits of detection and of quantification for transgenic sequences, determined by the SYBR Green PCR method developed by Jiang et al. [20], were 4 and 63 copies per reaction, respectively, for a viral vector, and 40 and 2560 copies per reaction for a plasmid template, respectively. In another study from this group, a different SYBR Green PCR method estimated the limit of detection at 2 and the limit of quantification at 16 and 32 copies (2 different primer sets) per reaction [26]. The assays sensitivity in these two studies was estimated in the absence of genomic DNA (i.e., competing template and, possibly, matrix impurities) and it is also difficult to directly compare this result with our test sensitivity due to the different accuracy of methods for quantification of vectors used for spiking, which was dPCR in our study, quantitative dot blot for viral vectors in [20,26], and spectrophotometry for the plasmid in [20].

Our method showed similar detection sensitivity to previously published assays when the transgenes were spiked into equine blood and extracted from plasma. All five transgenes spiked at 1500 cp/mL of blood and extracted with plasma (the concentration of doping material in the DNA extract is expected to be ~100–120 cp/µL) were detected by the screening and confirmation assays in this study. In [20,26], viral vectors spiked at 1000 cp/mL plasma and extracted using a different kit with the calculated concentration of the doping material in DNA extract of ~70 copies/µL were also detected. A lower amount of spiked EPO doping material (>130 copies/mL plasma) was detected in [18] by the dPCR method. Considering the similar detection sensitivities of the PCR assays used in both studies (~10 cp per reaction), our method may detect similar amount of spiked material in plasma, and we plan to test this in further studies.

Of crucial importance to the applicability of the developed test to doping control in horseracing are recent findings from several groups that tested their equine gene doping methods to detect the transgenic sequences administered to horses. Tozaki et al. detected the *EPO* transgene in the plasma up to two days following intramuscular administration of the equine EPO cDNA cloned into a plasmid using microfluidic real-time PCR and digital PCR methods [18,19]. The test developed by Cheung et al. detected the human EPO transgene in plasma up to eleven days and up to four weeks in blood following intramuscular administration of an rAAV vector carrying the transgene [7]. rAAV-specific sequences were also detected for up to 4 days in plasma and up to 28 days in synovial fluid following intra-articular administration [20]. These recent studies in horses complement the results of prolonged (in some cases, for weeks and even months) detection of transgenic sequences administered using rAAV or pDNA to mice, cynomolgus macaques, and to humans (reviewed in [8]). Since the test developed in this study has a similar detection sensitivity to the methods applied for the in vivo studies mentioned above, it is feasible that gene doping in racing horses will be detectable using our method for regulatory testing purposes. In further studies, we plan to extend the developed test using whole blood rather than plasma for DNA extraction to further increase the likelihood of gene doping detection. This is based on the prolonged detection time in blood or blood cells compared to plasma or serum shown in some studies (reviewed in [8]) with rAAV-mediated gene delivery, likely due to the transduction of blood cells with rAAV.

Further method development may also include an investigation of the possibility of extending confirmatory approaches to verify positive test results beyond FSA. These may include melt curve analysis by adding an intercalating dye compatible with a hydrolysis probe-labelling fluorophore to the PCR mix [36] or direct sequencing, possibly using tailed primers to increase the size of the PCR products or following cloning of the amplicon.

## 4. Materials and Methods

### 4.1. Materials

Five pUC19 (minus a multiple cloning site) plasmids, each containing a cloned cDNA for one of the five targeted genes, were supplied by Blue Heron Biotech (Bothell, WA, USA); the transgene sequence was confirmed by Sanger sequencing. A small volume of each plasmid was provided to the Viral Vector Production Unit (UPV) at the Universitat Autonoma Barcelona (Spain) to produce and supply five rAAV serotype 2/1 vectors, each carrying one equine transgene. The UPV purification procedures included benzonase and DNase I treatment, followed by precipitation with polyethylene glycol and iodixanol gradient ultracentrifugation. Certificates of analysis confirmed each transgene sequence identity and the high purity of the rAAV preparations.

High-performance liquid chromatography purified primers and hydrolysis (TaqMan) probes were purchased from Sigma (Merck, Darmstadt, Germany). Equine tissue genomic DNA (egDNA) extracted from a quarter horse was purchased from BioChain (Newark, CA, USA, Cat. No. D1034999). Brilliant II qPCR master mix (Cat. No. 600804) and Brilliant II SYBR Green qPCR master mix with 30 nM ROX (Cat. No. 600829) were from Agilent Technologies, Santa Clara, CA, USA. Qiagen qPCR multiplex master mix (Cat No. 206143), a QIAmp DNA blood midi kit (spin protocol, Cat. No. 51185), and a DyeEx 2.0 Spin kit (Cat. No. 63204) were from Qiagen GmbH (Hilden, Germany). AmpErase Uracil-N glycosylase (UNG) (Cat. No. N8080096) and a BigDye Terminator kit v3.1 (Cat. No. 4337457) were from Thermo Fisher Scientific, Waltham, MA, USA. All restriction enzymes were supplied by Genesearch (Arundel, QLD, Australia). A DNA 1000 kit (Agilent, Cat. No. 5067-1504) was used for fragment size analysis on Agilent 2100 Bioanalyzer. A pUC19 ladder and a Lamda HindIII ladder were from Thermo Fisher Scientific (Cat. No. SM0223 and SM0102, respectively).

Unless specified, all DNA dilutions were prepared in Tris-EDTA buffer (10 mM Tris, 0.1 mM EDTA, pH 8.0, further referred to as TE_0.1_). All other reagents were of molecular biology grade or required purity.

### 4.2. PCR Assay Design

The reference sequences for the five candidate doping genes (*EPO*, NM_001081825; *FST*, NM_001081811; *GH1*, NM_001081948; *IGF1*, NM_001082498; *IL1RN*, NM_001082525) were obtained from the horse reference genome EquCab2.1 in the GenBank database (https://www.ncbi.nlm.nih.gov/genbank/, accessed on 8 March 2016) and later cross-referenced against the upgraded release of EquCab3 [37]. Assay design was performed using Beacon Designer software v. 8.14 (PREMIER Biosoft, San Francisco, CA, USA). For the screening stage of the test, our intention was to design assays that could be multiplexed to simultaneously target several doping genes in a single analysis of a sample. For that, the software feature for design of multiplex assays was activated. For the confirmation stage, both design approaches (simplex and multiplex assay design) were used.

To quantify egDNA, an assay that targets the endogenous *EPO* gene was designed. The forward (5′-TCCAGACACCAAGGTTAA-3′) and reverse (5′-GAGACTCTCCAAGAGGAA-3′) primers in this assay target exon 2 and intron 2, respectively, generating an amplicon of 78 bp, while the probe (5′-FAM-AACTCACCTCCATCCTCTTCCAG-BHQ1-3′) anneals to the exon 2–intron 2 junction.

The probes for screening assays were labelled with unique fluorophores (Table 2) selected to avoid crosstalk between detection channels on the available PCR instrument. The probes for confirmation assays were labelled with FAM.

### 4.3. Accurate Quantification of the Materials Used for the In Vitro Gene Doping Model System

egDNA, five pDNA, and five rAAV preparations were accurately quantified by droplet dPCR (ddPCR) using a QX100 Droplet Digital PCR system (Bio-Rad Laboratories Pty. Ltd., South Granville, NSW, Australia) and a calibrated C1000 thermal cycler (Bio-Rad Laboratories Pty. Ltd., South Granville, NSW, Australia) essentially as described in Pinheiro et al. [38]. egDNA was quantified using the endogenous EPO gene assay described above. For pDNA and rAAV quantification, a pUC19 backbone-specific assay [39] and an assay targeting the cytomegalovirus promoter in the rAAV construct [40] were used, respectively. To improve ddPCR analysis, egDNA and circular pDNAs were subjected to restriction digest with MspI (the assays’ targets do not feature the MspI recognition site) using the previously described protocol [39]; successful digestion of both templates was confirmed by gel electrophoresis. Each material was analyzed in at least three sub-samples and three or four technical replicates.

### 4.4. Real-Time PCR Optimization and Analysis

All PCRs were performed in 96-well plates, with each reaction taking place in a single well.

PCR assay optimization was first performed using pDNA carrying the corresponding transgene in TE_0.1_ at 480 cp/well. The optimal primers concentration was determined in Brilliant II SYBR Green master mix; optimization of probe concentration for each assay was performed in Brilliant II qPCR master mix. For each assay, the temperature and duration of the annealing/extension step were optimized.

The next step of assay optimization was performed in a background of egDNA (100 ng/well), since in a real doping scenario, DNA extract from a sample will include genomic DNA as well as doping genetic material. Using 1 mL plasma from 2 mL blood samples obtained from several horses to extract DNA with the Qiagen DNA Blood Midi Kit, we established that the average concentration of egDNA in the DNA extracts was 25 ng/μL. Similar concentrations in DNA extracts from equine plasma using different extraction methods were measured in two other studies: 8.4 ± 10.4 ng/μL from over 1691 thoroughbred racehorses in [19] and 23.20 ± 10.85 ng/μL in [7]. With 4 μL of a DNA extract used in PCR in our study, the amount of egDNA per PCR well is expected to be around 100 ng.

Assay specificity was assessed using melt curve analysis following PCR performed in Brilliant II SYBR Green master mix and by analyzing the amplicon size using FSA on 4% agarose gel electrophoresis with a pUC19 ladder. To obtain more accurate fragment sizes, a Bioanalyzer with DNA 1000 kit was used as per the manufacturer’s instructions. Assay specificity was also assessed through the analysis of negative controls, NTC, where nuclease-free water or TE_0.1_ was used instead of DNA, and NegTC containing egDNA at 100 ng/well. The identity of the generated amplicons was confirmed by sequencing on an ABI 3500 Genetic Analyzer using a BigDye Terminator kit v3.1 and DyeEx 2.0 Spin kit to clean up the sequencing product. The generated sequence was analyzed using the BLASTn function on the NCBI website [41] with the Equus caballus genome as a reference.

Amplification efficiency and linearity in each assay were assessed by standard curve analysis using five 10-fold serial dilutions of the relevant pDNA or rAAV. Double-stranded pDNA was used in the concentration range between 10 and 10^5^ cp/well first in the absence of and subsequently in the presence of egDNA (100 ng/well). The standard curve analysis with single-stranded rAAV was performed within the concentration range between 20 and 2 × 10^5^ cp/well only with egDNA (100 ng/well). For both vectors, standard curve analysis was performed with and without restriction enzyme (RE).

In PCR, a 20 µL reaction contained 10 µL of 2× master mix, 1 µL of 20× assay primer and probe mix, and 0.2 µL (1 U/µL) of UNG to eliminate carryover contamination. Where required, a RE was added at the optimized concentration (described in the Section 2). The remaining volume was occupied by solution(s) of DNA (pDNA or rAAV, with or without background egDNA, or a DNA extract) and nuclease-free water. NTC and, where relevant (e.g., in assay optimization and testing assay specificity), NegTC were analyzed in parallel. All samples and controls were analyzed in duplicate.

The standard thermal profile consisted of an UNG incubation at 50 °C for 2 min, polymerase activation and template denaturation at 95 °C for 15 min (Qiagen Multiplex MM) or 10 min (Brilliant II MM), followed by 45 cycles of template denaturation at 95 °C for 30 s, and a combined annealing/extension step for 30 s. The optimized annealing/extension temperature for each assay is reported in the Section 2. For assays that included an RE step (all screening and EPO, FST, and GH1 confirmation), thermocycling was preceded by a 60 min incubation at 37 °C.

The gain setting for the Cy3 channel was two, while for all other detection channels it was one. Following assay optimization, the fluorescence threshold for each assay (dR, baseline corrected raw fluorescence) was validated and fixed. For the screening assays, it was 100 units for the EPO, FST, and GH1 assays, 300 for IGF1, and 150 for IL1RN. For the confirmation assays, the threshold was 250 units for EPO, 300 for FST and GH1, and 200 for IGF1 and IL1RN.

Most real-time PCR work was performed using a MX3005P PCR machine (Agilent). Optimization of the annealing/extension temperature was performed using a Step One Plus Real-Time PCR System (Applied Biosystems, Foster City, CA, USA).

### 4.5. Restriction Enzyme Digestion in Real-Time PCR

To find a suitable RE to improve real-time PCR performance in the presence of egDNA, we used NEB Cutter V2.0 (nc2.neb.com/NEBcutter2, accessed on 22 September 2016) to target a restriction site in the intron sequence framed by exons targeted by primers. To allow the screening assays to be performed in multiplex, a single RE was sought for which the five amplicons generated in these assays did not feature restriction sites. To test the effectiveness of the selected REs in the PCR master mix, digestion reactions were performed in the relevant 1× PCR master mix instead of NEB buffer, followed by gel electrophoresis using 1% agarose gel with a Lamda HindIII ladder.

### 4.6. Multiplexing of the Screening Assays

The screening assays optimized in simplex were tested in duplex (EPO and GH1), triplex (FST, IGF1, and IL1RN), four-plex (EPO, FST, IGF1, and IL1RN) and five-plex formats using pDNA carrying the target transgene (one pDNA at a time). For this, depending on the combination, individual 20X primer–probe mixes were added to the reaction mix at the expense of nuclease-free water. The performance of each assay in multiplex was assessed by standard curve analysis similarly to as described above for the simplex format. Results were compared to those generated by the assays performed in simplex. The multiplex format that showed the best performance for each screening assay with pDNA was identified and then assessed with rAAV vectors.

### 4.7. Samples for Competency and Surveillance Gene Doping Testing

Whole blood samples were sourced from the Thoroughbred racehorse population by Racing NSW under ethics approval from the Racing NSW Animal Care and Ethics Committee (Racing NSW Animal Research Authority 69 ‘Surveillance study for the detection of gene doping in thoroughbred racehorses’).

For the competency study, whole blood (8–10 mL) was collected in lithium heparin vacutainers (Becton Dickinson, Mississauga ON, Canada, Cat. No. 367880) from the jugular veins of 14 horses, following the demonstration that the test performed similarly whether using plasma prepared from these or EDTA-containing vacutainers (Becton Dickinson, Mississauga, ON, Canada, Cat. No. 366643). This is the routine blood collection procedure at Racing NSW. Ten 4 mL aliquots of each blood sample were spiked with one of the five pDNA at 1500 cp/mL or one of the five rAAV at 1500 vg/mL. This amount of spiked doping material was chosen to give a clear positive result in the test, as it should result in ~120 cp of transgene per PCR well. This amount was calculated based on the experimental conditions followed (1.5 mL of plasma was used in DNA extraction, the average volume of eluted DNA is close to 150 µL and 4 µL of DNA extract is used in PCR) and the assumptions that plasma constitutes 50% of blood, all spiked doping material distributes to plasma, and the extraction recovery is 100%. In total, for each transgene, two mock positive blood samples were made. Four 4 mL aliquots of remaining samples were not spiked and served as negative samples for gene doping. The analyst had no knowledge of which samples had been spiked or which doping gene and vector had been used. For practical reasons, the samples were divided into two batches for processing and analysis (Samples 1–7 in Batch 1, and Samples 8–14 in Batch 2).

For surveillance testing, samples from 18 horses were received for gene doping testing on three separate dates as part of standard out of competition doping surveillance carried out by Stipendiary Stewards. The number of samples received on any given day (a batch) was capped at six. Blood samples for each horse were collected in 10 mL lithium heparin tubes, transported and stored at 4 °C. The samples were equilibrated to room temperature before analysis. The time between blood collection and DNA extraction varied between one (Batch 2 and Batch 3) and four days (Batch 1).

In both studies, if blood started separating into plasma and cells, the samples were homogenized by gentle inversion eight times and centrifuged (Eppendorf 5810R with a swing-out rotor; Eppendorf AG, Hamburg, Germany) at 1000× *g* for 10 min at room temperature. Plasma (1.5 mL) was mixed with 0.5 mL of phosphate-buffered saline, and DNA was extracted using the QIAmp DNA blood midi kit following the manufacturer’s protocol for a 2 mL sample with reloading of the primary DNA eluate on the column for extra purification. PEC and NEC were prepared and extracted with each batch of samples. For NEC, 1 mL of TE_0.1_ was extracted to monitor any possible contamination arising from the extraction process. PEC consisted of 1 mL of TE_0.1_ spiked with 750 cp of pDNA carrying the IL1RN transgene. Both controls were extracted using the QIAmp DNA blood midi kit protocol for 1 mL sample volume.

PCR was performed within 24 h of extraction in parallel with PCR and extraction controls, and the inhibition control for each sample. PEC extract was analyzed using the screening IL1RN assay in simplex.

## 5. Conclusions

In this study we have developed and validated a set of PCR assays to detect five transgenes, namely EPO, FST, GH1, IGF1, and ILRN1, in equine anti-doping samples. The assays showed excellent specificity and high sensitivity that was comparable to that in methods being developed in other jurisdictions. The novel use of a pre-PCR restriction digestion to improve assay specificity and sensitivity was introduced in a way that maintains the integrity of the sample, ease of workflow, and reliability of doping genes detection. The assays formed the basis of a two-stage test protocol, with the screening and the confirmation stages relying on two different transgene-specific assays that target different sections within the doping gene. The two-step test protocol allows the gene doping test to better withstand legal scrutiny and has already been approved for gene doping detection in human athletes [30]. We simplified screening for the five doping genes by developing the PCR protocol, where all five genes can be tested for in one PCR analysis, with four genes being screened for simultaneously in one PCR reaction. This makes the test faster and more economical. Having been tested in equine blood samples and having been demonstrated to be fit for purpose, this test will be introduced into the suite of tests used to detect prohibited substances in racehorses in Australia.

The test developed in this study and other published methods that provide highly sensitive and specific transgene detection, together with the body of evidence that injected genetic material can persist for days, weeks and, sometimes months in bodily fluids of racing horses, humans, nonhuman primates, and other animals, provides confidence that the new illegal approach to enhance performance using genetic manipulations will be detected and punished. This will help to maintain fair play and the integrity of horseracing and will protect the welfare of equine athletes.

## Figures and Tables

**Table 1:** Nucleotide sequences of *GH1* assays.

Oligo Positions in Transgene	Sequence (5′ to 3′)
Screening assay F3-P3/4-R4	F: TGCCTTCTGCTTCTC
	R: CCACGACTGGATGAG
	P: AGCTCCATGTCAGATCTCTGCTG
Confirmation assay F2-P2/3-R3	F: ATGCCGTTGTCTAGC
	R: CGAGTATCTCTGTCCC
	P: CGCGCTCAAACTCTTTGTAG

Oligo position in each transgene refers to the exon or exon–exon junction in which the primer or probe sits. The oligo spanning the exon–exon junction is underlined. Abbreviations: F, forward primer; P, probe; R, reverse primer.

**Table 2:** PCR assays designed to detect five equine doping genes; sizes of amplicon generated from transgenes and their corresponding endogenous genes, and fluorophores used to label assays’ hydrolysis probes.

Assay	Fluorophore	Amplicon Size (bp)	
		from cDNA	from egDNA
EPO (s)	FAM	100	>300
FST (s)	JOE	142	>800
GH1 (s)	Cy3	109	308
IGF1 (s)	TxRed	127	>1000
IL1RN (s)	Cy5	149	>1000
EPO (c)	FAM	113	>300
FST (c)	FAM	112	>500
GH1 (c)	FAM	120	333
IGF1 (c)	FAM	97	>1000
IL1RN (c)	FAM	199	No product

The last column shows the approximate size of a hypothetical intron-spanning amplicon from the corresponding endogenous gene in equine genomic DNA. Only the approximate size is indicated for most assays to prevent disclosing information that could be used to identify the target boundaries. This information could then be used to manipulate the transgene to evade detection. ‘No product’ is noted for the IL1RN confirmation assay because a primer spans the exon–exon junction, preventing amplification from egDNA. Abbreviations: (s) and (c) refer to PCR assays assigned for the screening or confirmation stages of the gene doping test, respectively; bp, base pairs; cDNA, coding DNA for the gene; egDNA, equine genomic DNA.

**Table 3:** Minimum amount of transgene reliably (in ≥95% replicates) detectable in the screening and confirmation assays performed in simplex in the presence of 100 ng of egDNA per PCR well.

	pDNA		rAAV	
	Sensitivity	Repeatability	Sensitivity	Repeatability
Assay	cp/well	Replicates Detected ^a^	vg/well	Replicates Detected ^a^
*EPO* (s)	20	15/16 (95%)	20	16/16 (100%)
*FST* (s)	10	18/18 (100%)	10	15/16 (95%)
*GH1* (s)	10	16/16 (100%)	20	15/16 (95%)
*IGF1* (s)	10	16/16 (100%)	10	16/16 (100%)
*IL1RN* (s)	10	16/16 (100%)	10	15/16 (95%)
*EPO* (c)	10	16/16 (100%)	10	16/16 (100%)
*FST* (c)	10	15/16 (95%)	10	16/16 (100%)
*GH1* (c)	10	15/16 (95%)	10	16/16 (100%)
*IGF1* (c)	10	15/16 (95%)	10	16/16 (100%)
*IL1RN* (c)	10	16/16 (100%)	15	16/16 (100%)

^a^ ‘Replicates detected’ shows the number of replicates detected as positive out of the total number of replicates tested. The percentage of detected replicates is shown in parenthesis and is rounded to the nearest multiple of five. The accuracy of the sensitivity values is reliant on accurate dPCR-based quantification of each vector used. Copy numbers less than ten were not tested. Abbreviations: (s), screening assay; (c), confirmation assay; cp, copies of pDNA; vg, rAAV viral genome.

**Table 4:** Minimum amount of transgene reliably (in ≥95% of replicates) detectable in the four-plex combination of screening assays for the EPO, FST, IGF1, and IL1RN transgenes performed in the presence of 100 ng of egDNA per PCR well.

Assay	Template	Sensitivity (cp or vg per well)	Repeatability (Replicates Detected) ^a^
*EPO*	pDNA	20	15/16 (95%)
	rAAV	40	16/16 (100%)
*FST*	pDNA	10	16/16 (100%)
	rAAV	20	16/16 (100%)
*IGF1*	pDNA	10	16/16 (100%)
	rAAV	20	16/16 (100%)
*IL1RN*	pDNA	10	16/16 (100%)
	rAAV	20	15/16 (95%)

^a^ ‘Replicates detected’ shows the number of replicates detected as positive out of the total number of replicates tested. The percentage of detected replicates is shown in parenthesis and is rounded to the nearest multiple of five. Copy numbers less than ten were not tested. Abbreviations: cp, copies of pDNA; vg, viral genome for rAAV.

**Table 5:** Results from blind testing of samples using the screening assays.

Sample Name	EPO			FST			GH1			IGF1			IL1RN		
	PCR	IC	Finding	PCR	IC	Finding	PCR	IC	Finding	PCR	IC	Finding	PCR	IC	Finding
1	−/−	***41.0 (6.8)***	Neg	−/−	30.5 (1.5)	Neg	−/−	37.01 (4.0)	Neg	−/−	30.9 (1.2)	Neg	−/−	30.0 (1.2)	Neg
2	−/−	***40.4 (6.2)***	**Neg**	−/−	30.3 (1.3)	Neg	−/−	36.8 (3.8)	Neg	−/−	30.5 (0.8)	Neg	−/−	30.0 (1.2)	Neg
3	−/−	***40.6 (6.5)***	Neg	−/−	29.9 (0.9)	Neg	−/−	35.4 (2.4)	Neg	−/−	30.8 (1.1)	Neg	−/−	29.8 (1.0)	Neg
4	−/−	***42.5 (8.4)***	Neg	−/−	30.0 (1.0)	Neg	−/−	35.5 (2.5)	Neg	−/−	30.4 (0.6)	Neg	+/+	29.7 (1.0)	**Pos**
5	−/−	***No Ct***	Neg	−/−	31.1 (2.1)	Neg	−/−	37.4 (4.4)	Neg	+/+	30.9 (1.1)	**Pos**	−/−	30.8 (2.0)	Neg
6	−/−	***42.9 (7.9)***	Neg	−/−	30.8 (1.8)	Neg	−/+	37.8 (4.7)	**Pos**	−/−	31.0 (1.2)	Neg	−/−	31.8 (3.0)	Neg
7	−/−	***No Ct***	Neg	+/+	30.6 (1.6)	**Pos**	−/−	36.6 (2.8)	Neg	−/−	30.4 (0.6)	Neg	−/−	30.1 (1.3)	Neg
8	−/−	***40.2 (6.0)***	Neg	−/−	31.0 (2.0)	Neg	−/−	37.3 (3.5)	Neg	−/−	30.9 (1.2)	Neg	−/−	30.5 (1.8)	Neg
9	+/+	37.6 (3.8)	**Pos**	−/−	30.5 (1.5)	Neg	−/−	36.8 (3.0)	Neg	−/−	30.6 (0.8)	Neg	−/−	30.2 (1.4)	Neg
10	−/−	38.1 (3.9)	Neg	−/−	30.6 (1.6)	Neg	−/−	35.9 (2.1)	Neg	−/−	30.3 (0.5)	Neg	+/+	30.1 (1.3)	**Pos**
11	−/−	37.9 (3.8)	Neg	−/−	30.5 (1.5)	Neg	−/−	36.1 (2.2)	Neg	−/−	30.6 (0.8)	Neg	−/−	30.0 (1.2)	Neg
12	−/−	39.2 (5.0)	Neg	−/−	30.0 (1.0)	Neg	−/−	35.5 (1.7)	Neg	+/+	30.3 (0.6)	**Pos**	−/−	30.3 (1.5)	Neg
13	−/−	***42.6 (8.4)***	Neg	−/−	30.9 (1.8)	Neg	+/+	38.0 (4.2)	**Pos**	−/−	30.7 (1.0)	Neg	−/−	30.3 (1.5)	Neg
14	−/−	***42.8 (8.6)***	Neg	+/+	30.0 (0.9)	**Pos**	−/−	36.0 (2.1)	Neg	−/−	30.8 (1.0)	Neg	−/−	30.2 (1.4)	Neg
NEC	−/−		Pass	−/−		Pass	−/−		Pass	−/−		Pass	−/−		Pass
NTC	−/−		Pass	−/−		Pass	−/−		Pass	−/−		Pass	−/−		Pass
PEC	N/A		N/A	N/A		N/A	N/A		N/A	N/A		Pass	+/+		Pass
PTC	+/+		Pass	+/+		Pass	+/+		Pass	+/+		Pass	+/+		Pass

Preparation of blood samples from 14 horses is described in Section 4.7. Mock positive samples were spiked with a single transgene. For each transgene, two positive samples were made, one with pDNA and another with rAAV, both vectors carrying the specific transgene. Four samples (1, 3, 8, and 11) remained un-spiked. The samples were analyzed in two batches: testing for EPO, FST, and GH1 was performed in Batch 1, while testing for IGF1 and IL1RN was performed in Batch 2. ‘PCR’ column shows the PCR result (+ and − indicating a positive or a negative result, respectively) for each replicate reported separately. The inhibition control (IC) results show the average Ct value for IC and, in brackets, delta Ct between the IC and PTC. The IC values shown in bold italics indicate significant PCR inhibition as per the test protocol acceptance criteria described in the text (Section 2.6). N/A indicates ‘not analyzed’, as PEC is only analyzed in the IL1RN assay (refer to Section 4.7). Neg refers to the negative result for the doping gene, Neg in bold indicates a false-negative result, i.e., the sample had been spiked with the transgene, but was PCR negative. Pos in bold refers to the sample been correctly reported positive for the doping gene. These positive results were verified by the corresponding confirmation assay. The average (*n* = 2) Ct values for positive results were as follows: EPO—41 (sample 9); FST—35 and 32 (samples 7 and 14); GH1—44 and 43 (samples 6 and 13); IGF1—36 and 34 (samples 5 and 12); IL1RN—31 and 33 (samples 4 and 10). Abbreviations: IC, inhibition control; NEC, negative extraction control; NTC, no template control; PEC, positive extraction control; PTC, positive template control.

**Table 6:** PCR results in the screening assays for Batch 3 of equine blood samples submitted for official doping control and used for surveillance gene doping testing.

Sample	EPO		FST		GH1		IGF1		IL1RN	
	PCR	IC	PCR	IC	PCR	IC	PCR	IC	PCR	IC
1	−/−	36.3 (4.5)	−/−	32.1 (2.3)	−/−	***43.6 (9.7)***	−/−	35.2 (4.9)	−/−	39.0 (5.7)
2	−/−	36.5 (3.7)	−/−	31.3 (1.5)	−/−	38.2 (4.4)	−/−	31.4 (1.1)	−/−	31.1 (1.6)
3	−/−	36.2 (3.4)	−/−	29.5 (0.3)	−/−	38.8 (4.9)	−/−	31.5 (1.2)	−/−	30.2 (0.7)
4	−/−	35.4 (3.6)	−/−	30.1 (0.3)	−/−	39.3 (5.4)	−/−	32.0 (1.7)	−/−	31.4 (1.9)
5	−/−	35.6 (4.1)	−/−	31.1 (1.4)	−/−	***40.1 (6.3)***	−/−	31.7 (1.4)	−/−	30.7 (1.2)
6	−/−	35.9 (5.1)	−/−	30.0 (0.2)	−/−	38.4 (4.5)	−/−	31.7 (1.4)	−/−	31.2 (1.7)
NEC	−/−	Pass	−/−	Pass	−/−	Pass	−/−	Pass	−/−	Pass
NTC	−/−	Pass	−/−	Pass	−/−	Pass	−/−	Pass	−/−	Pass
PEC	N/A		N/A		N/A		N/A		+/+	Pass
PTC	+/+	Pass	+/+	Pass	+/+	Pass	+/+	Pass	+/+	Pass

‘PCR’ column shows the PCR result (+ and − indicating a positive or a negative result, respectively) for each replicate. The inhibition control (IC) results show the average Ct value for IC and, in brackets, delta Ct between the IC and PTC. The IC values shown in bold italics indicate significant PCR inhibition as per the test protocol acceptance criteria. N/A indicates ‘not analyzed’, as PEC is analyzed only in the IL1RN assay (refer to Section 4.7).

## Data Availability

The sequences of all but one of the assays’ oligonucleotides are not presented here to preserve the integrity of the gene doping detection test but can be made available on request to the corresponding author after signing a non-disclosure agreement.

## Supplementary Materials

The supporting information can be downloaded at: https://www.mdpi.com/article/10.3390/ijms25052570/s1.
