# Supplementary material for: PCR-Based Equine Gene Doping Test for the Australian Horseracing Industry
Source: Int J Mol Sci. 2024 Feb 22;25(5):2570. doi: 10.3390/ijms25052570 (PMC10931823; doi:10.3390/ijms25052570)
Supplement: Supplementary file 1 [file ijms-25-02570-s001.zip › ijms-2840039-supplementary.pdf]

SUPPLEMENTARY RESULTS

Figure S1. Assay sensitivity in the presence of varying amounts of egDNA. Ct values for each transgene (160 copies pDNA /well) are shown in their corresponding screening and confirmation PCR assays in the presence of varying amounts of egDNA.

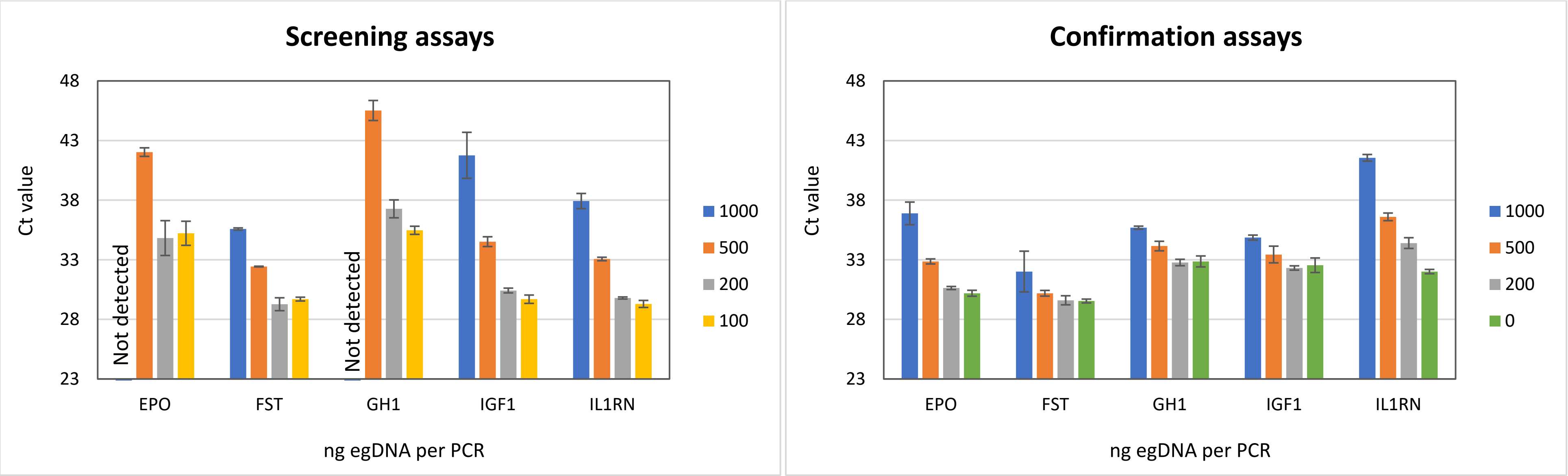

Each condition was analysed in duplicate, with each bar showing the average Ct value and error bars representing the deviation between individual Ct values. “Not detected” means that both replicates were PCR negative. For each assay, the fluorescence threshold was manually determined and validated (refer to Materials and Methods). Since prior to this experiment, we established that Ct values for all transgenes in their screening assays in the presence or absence of egDNA at 100 ng/well were similar, in this experiment, for practical reasons, we did not use the condition with no egDNA in the screening assays. For the same reason, in the experiment with the confirmation assays, the condition of egDNA at 100 ng/well was omitted.

Table S1. Performance parameters of the screening and confirmation assays in simplex using the corresponding plasmid carrying the transgene as template.

	Screening assays							Confirmation assays			
	Reaction conditions	Brilliant II qPCR Master Mix			Qiagen Multiplex Master Mix			Qiagen Multiplex Master Mix			
Transgene		Efficiency (%)	Linearity	Ct avg (Half range) (10 cp/well)	Efficiency (%)	Linearity	Ct avg (Half range) (10 cp/well)	Reaction conditions	Efficiency (%)	Linearity	Ct avg (Half range) (10 cp/well)
EPO	$TE_{0.1}$	99	0.98	40.0 (0.9)	99	0.99	38.0 (0.7)	$TE_{0.1}$	96	0.99	37.0 (0.6)
	egDNA	84*	0.94*	No Ct	108*	0.97*	39.5	egDNA	85^	0.91	No Ct
	egDNA+BanII	75	0.95	43.1 (1.3)	99	1	34.7 (0.3)	egDNA+MspI	100	1	34.6 (0.1)
FST	$TE_{0.1}$	97	0.95	39.4 (1.7)	97	0.99	36.3 (0.7)	$TE_{0.1}$	101	1	35.4 (0.2)
	egDNA	97	1	39.8 (0.1)	84	0.98	37.9 (1.4)	egDNA	96	0.99	36.3 (0.2)
	egDNA+BanII	100	0.99	38.1 (0.4)	100	0.98	32.9 (0.3)	egDNA+BanII	103	1	32.8 (0.2)
GH1	$TE_{0.1}$	97	1	38.9 (0.6)	101	0.96	36.0 (1.9)	$TE_{0.1}$	100	0.99	36.5 (0.6)
	egDNA	124	0.84	44.1 (0.8)	89	0.99	37.4 (0.1)	egDNA	69*	0.98	44.8
	egDNA+BanII	99	1	37.8 (0.3)	94	1	36.0 (0.03)	egDNA+EcoRI	92	0.99	36.7 (0.2)
IGF1	$TE_{0.1}$	103	1	36.6 (0.5)	99	1	34.4 (0.8)	$TE_{0.1}$	102	0.99	37.9 (0.7)
	egDNA	103	0.98	39.3 (1.4)	92	0.99	34.7 (0.5)	egDNA	110	1	36.8 (0.3)
	egDNA+BanII	97	1	36.1 (0.1)	98	0.99	34.3 (0.1)				
IL1RN	$TE_{0.1}$	114	0.99	35.6 (0.8)	105	0.99	33.5 (0.5)	$TE_{0.1}$	97	0.97	38.4 (1.6)
	egDNA	113	0.99	37.3 (0.5)	102	0.95	34.2 (1.5)	egDNA	100	0.99	39.8 (0.8)
	egDNA+BanII	109	0.99	37.3 (0.5)	90	0.99	33.4 (0.8)				

Shown are the results from standard curve analyses using as template a corresponding plasmid within the dynamic range of five orders of magnitude (10 to 10^5 cp/well), Qiagen Multiplex master mix and optimized PCR conditions. Three reaction conditions were tested: in $TE_{0.1}$ and in the presence of equine genomic DNA (egDNA) at 100 ng/well without or with a suitable restriction enzyme (refer to the Results section in the paper), except for the IGF1 and IL1RN confirmation assays, where no restriction digest was used.

Ct avg (Half range) refers to the average Ct value and half range for duplicate measurements of the lowest plasmid dilution tested (10 cp/well). Where Half range is not shown, one of two replicates did not amplify.

* Indicates where only four dilutions ranging between 10^5 to 100 cp/well were used to calculate efficiency and linearity because the lowest dilution (10 cp/well) was not detected in one or both replicates.

^ Indicates where only three dilutions ranging between 10^5 to 10^3 cp/well were used to calculate efficiency and linearity because two lowest dilutions (10 and 100 cp/well) were not detected.

Figure S2. Fluorescence spectra for the five fluorophores (FAM, JOE, Cy3, Texas Red, and Cy5) used to label the probes in the screening assays.

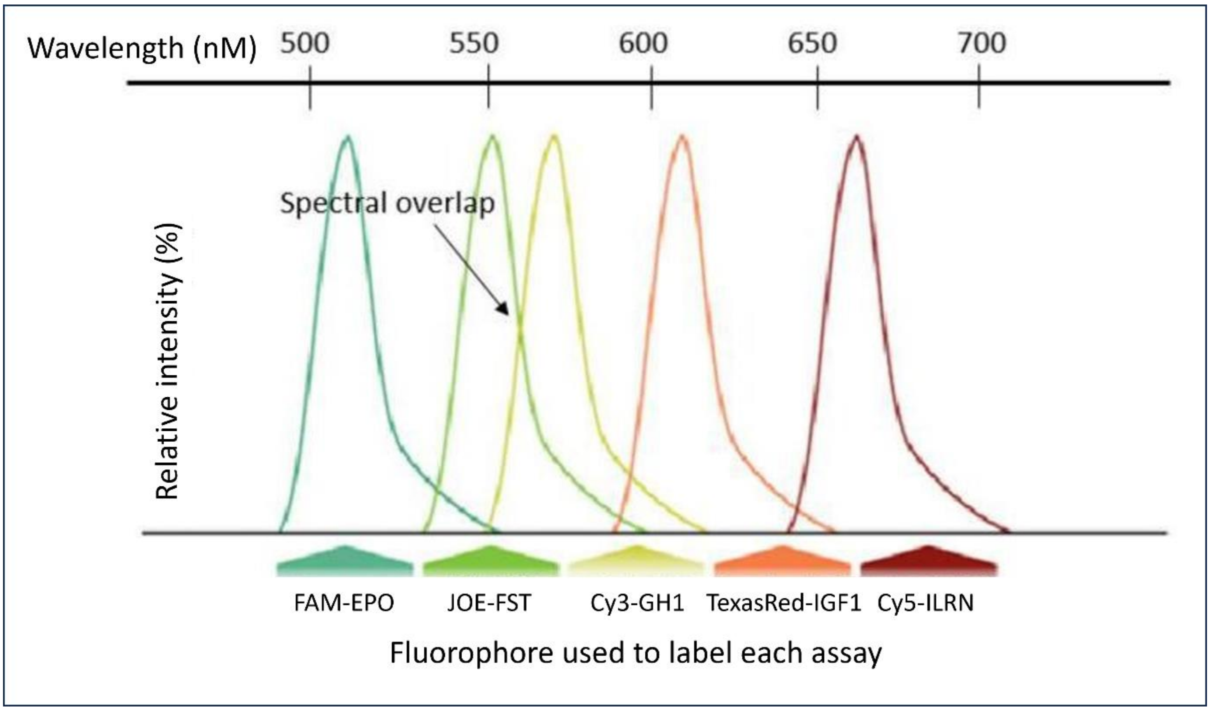

This figure shows considerable spectral overlap between JOE and Cy3 which alerts to the possibility of crosstalk between the two fluorophores when the GH1 (Cy3) and FST (JOE) assays are multiplexed.

Figure S3. Quality control chart for PTC for the EPO screening assay.

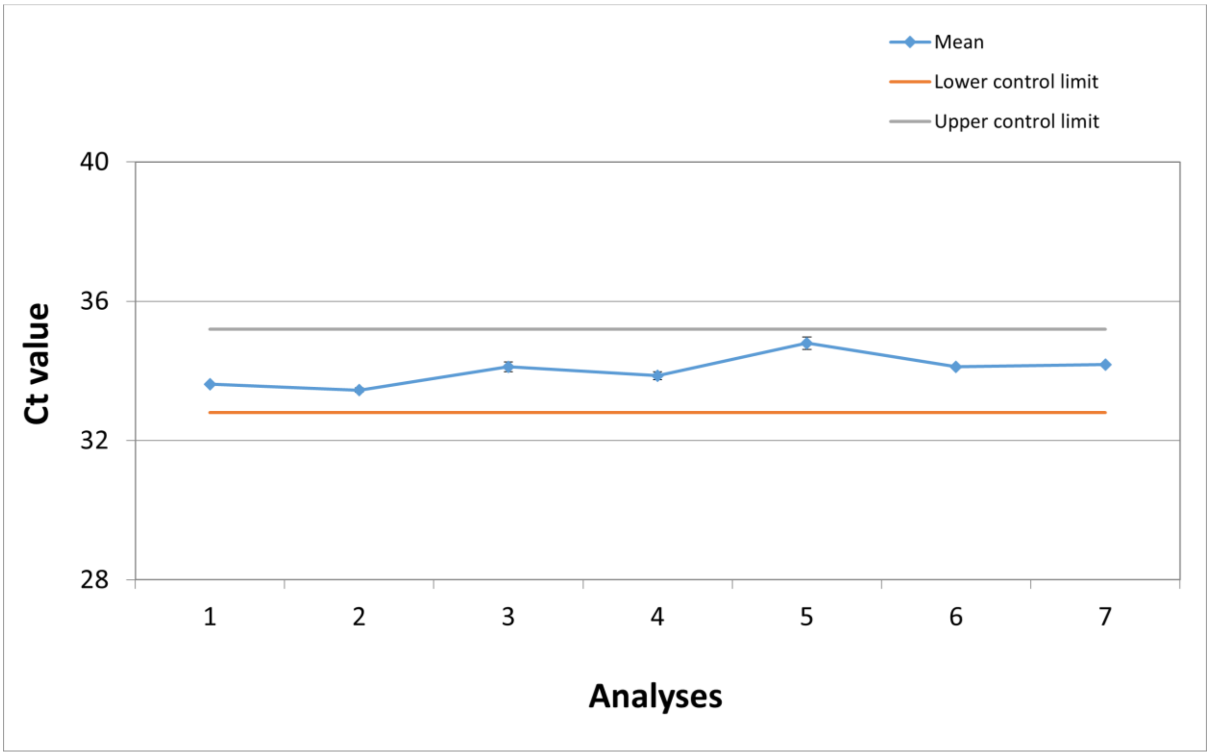

For each PTC analysis, an average Ct of duplicates is shown with deviation between replicates represented by error bars. Details on how quality control charts are created and how they monitor stability of the measurement system are described in the paper.

Table S2. Results for each screening assay performed on samples from two batches of equine blood samples obtained for surveillance testing.

Batch	Sample name	EPO		FST		GH1		IGF1		IL1RN	
		PCR result	IC result	PCR result	IC result	PCR result	IC result	PCR result	IC result	PCR result	IC result
1	B146870	-/-	37.3 (3.7)	-/-	31.3 (1.9)	-/-	37.3 (3.7)	-/-	31.3 (1.9)	-/-	37.3 (3.7)
	B144476	-/-	36.4 (3.8)	-/-	30.1 (0.7)	-/-	36.4 (3.8)	-/-	30.1 (0.7)	-/-	36.4 (3.8)
	B143402	-/-	37.4 (3.8)	-/-	30.3 (0.9)	-/-	37.4 (3.8)	-/-	30.3 (0.9)	-/-	37.4 (3.8)
	B146872	-/-	36.3 (4.7)	-/-	30.5 (1.1)	-/-	36.3 (4.7)	-/-	30.5 (1.1)	-/-	36.3 (4.7)
	B144650	-/-	36.3 (4.7)	-/-	30.1 (0.7)	-/-	36.3 (4.7)	-/-	30.1 (0.7)	-/-	36.3 (4.7)
	B145485	-/-	35.3 (4.7)	-/-	30.4 (1.0)	-/-	35.3 (4.7)	-/-	30.4 (1.0)	-/-	35.3 (4.7)
	NEC	-/-	Pass	-/-	Pass	-/-	Pass	-/-	Pass	-/-	Pass
	NTC	-/-	Pass	-/-	Pass	-/-	Pass	-/-	Pass	-/-	Pass
	PEC	N/A	N/A	N/A	N/A	N/A	N/A	N/A	N/A	+/+	Pass
	PTC	+/+	Pass	+/+	Pass	+/+	Pass	+/+	Pass	+/+	Pass
2	B143364	-/-	36.2 (2.8)	-/-	31.1 (2.3)	-/-	34.6 (3.2)	-/-	31.6 (1.7)	-/-	29.7 (0.6)
	B143591	-/-	37.6 (4.2)	-/-	30.0 (1.1)	-/-	35.3 (3.9)	-/-	31.2 (1.2)	-/-	29.5 (0.4)
	B144850	-/-	37.1 (3.7)	-/-	30.4 (1.5)	-/-	34.8 (2.5)	-/-	31.1 (1.1)	-/-	29.8 (0.8)
	B145496	-/-	36.3 (4.9)	-/-	30.5 (1.6)	-/-	34.2 (1.9)	-/-	31.1 (1.1)	-/-	30.3 (1.2)
	B142925	-/-	36.7 (3.2)	-/-	30.3 (1.4)	-/-	35.0 (3.7)	-/-	30.5 (0.5)	-/-	29.9 (0.9)
	B144164	-/-	36.0 (3.5)	-/-	30.1 (1.2)	-/-	34.7 (2.4)	-/-	31.1 (1.1)	-/-	30.7 (1.6)
	NEC	-/-	Pass	-/-	Pass	-/-	Pass	-/-	Pass	-/-	Pass
	NTC	-/-	Pass	-/-	Pass	-/-	Pass	-/-	Pass	-/-	Pass
	PEC	N/A	N/A	N/A	N/A	N/A	N/A	N/A	N/A	+/+	Pass
	PTC	+/+	Pass	+/+	Pass	+/+	Pass	+/+	Pass	+/+	Pass

In PCR results, + and – indicate, respectively, a positive and negative PCR result for each PCR replicate. The IC results show the average Ct value with delta Ct in brackets. N/A was recorded for assays that were not tested with the PEC, as it is only tested in the IL1RN screening assay. All controls passed and all samples were reported as negative.
